# Mask mandates in light of DANMASK-19

**DOI:** 10.1017/ice.2021.35

**Published:** 2021-01-29

**Authors:** Sajith Matthews

**Affiliations:** Division of General Medicine, Department of Internal Medicine, Wayne State University, Detroit, Michigan

*To the Editor*—When public pressure mounted for the use of hydroxychloroquine (HCQ) for prophylaxis or treatment of coronavirus disease 2019 (COVID-19), our nation’s leading scientists exercised prudence and recommended awaiting the results of randomized controlled trials (RCTs) before considering its use. Such restraint proved to be invaluable because evidence from these RCTs ultimately showed that there is no benefit but rather harm with HCQ use in the treatment of COVID- 19.^1,2^ A similar focus on high-quality evidence has not been taken for masks and effect on mitigating the spread of disease. Internationally, public health mandates for masks in the community, has varied from no masks to mandatory masks when outside in crowds to wearing masks when symptomatic.^3–5^ Acknowledging the lack of evidence from RCTs of masks having any additive effects on mitigating the transmission of severe acute respiratory syndrome coronavirus 2 (SARS-CoV-2),^5^ public mask use was recommended by the Center for Disease Control (CDC) for protective effect (among healthy individuals) and not just source control (among symptomatic individuals).

The DANMASK-19 was a well-powered randomized controlled trial (6,000 participants) with 46% proper and 47% predominantly proper adherence to mask use in a setting of uncommon mask use, moderate spread of infection, and reasonable adherence to social distancing and handwashing.^6^ The DANMASK-19 trial was consistent with the 12 previous RCTs^7^ which showed, with moderate certainty evidence, that there were negligible additive effects from masks in the prevention of respiratory infections. The DANMASK-19 trial showed the mask’s protective effect to be inconclusive and difference between the 2 groups to not be statistically significant in the community setting. Despite the evidence from previous RCTs on influenza and other respiratory viral infections, there was suspicion from observational studies^8^ that severe acute respiratory syndrome SARS-CoV-2 behaved differently and that droplet transmission could be mitigated by mask use in the presymptomatic phase.^5^ Therefore, the implementation of universal mask use was justified while awaiting the results DANMASK 19. In light of the inconclusive evidence from DANMASK 19 and the previous RCTs, the case for a protective effect from COVID 19 lacks evidence and requires modification from public health officials.

Although this study did not assess source control, the effect of masks is compelling, when restricted to contacts of index cases receiving the intervention within 36 hours of symptom onset.^9^ Hence, mask use among symptomatic individuals and their contacts is evidence based. On the contrary, long-term effects of mask use among healthy individuals is unknown,^3^ and short-term effects include breathing difficulties, self infection through touching eyes due to irritation from exhaled air from masks, and a false sense of security from mask while neglecting social distancing.^10^ The argument for masks having a variolation effect in COVID-19 is compelling,^11^ but it lacks the support of evidence from cohort studies. Hence, with the current data available, the best case for masks appears to be in symptomatic patients and recommended (not mandatory) use in crowded settings. Wisdom to use measured language in what we “mandate” and “recommend” would be advised. We must decide with prudence, as we did with HCQ, what we choose to be “absolutely essential” measures, and we must decide these based upon robust evidence. In the haste of establishing “life saving” measures, we may be instead be losing the public’s trust by not having the supportive evidence and unintentionally placing the lives of the community and healthcare workers at risk.
